# Supplementary material for: Streptococcus salivarius 24SMBc Genome Analysis Reveals New Biosynthetic Gene Clusters Involved in Antimicrobial Effects on Streptococcus pneumoniae and Streptococcus pyogenes
Source: Microorganisms. 2022 Oct 16;10(10):2042. doi: 10.3390/microorganisms10102042 (PMC9610097; doi:10.3390/microorganisms10102042)
Supplement: Supplementary file 1 [file microorganisms-10-02042-s001.zip › Figure S1.pdf]

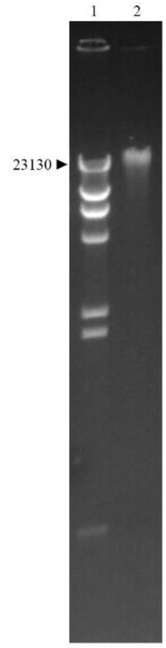

Figure S1. Agarose gel electrophoretic analysis of *S. salivarius 24SMBc* genomic DNA. Lane 1, Lambda DNA/HindIII (500 ng). Lane 2, genomic DNA extraction with PureLink™ Genomic DNA Mini Kit (1 µl).
