# Supplementary material for: Streptococcus salivarius 24SMBc Genome Analysis Reveals New Biosynthetic Gene Clusters Involved in Antimicrobial Effects on Streptococcus pneumoniae and Streptococcus pyogenes
Source: Microorganisms. 2022 Oct 16;10(10):2042. doi: 10.3390/microorganisms10102042 (PMC9610097; doi:10.3390/microorganisms10102042)
Supplement: Supplementary file 1 [file microorganisms-10-02042-s001.zip › Figure S2.pdf]

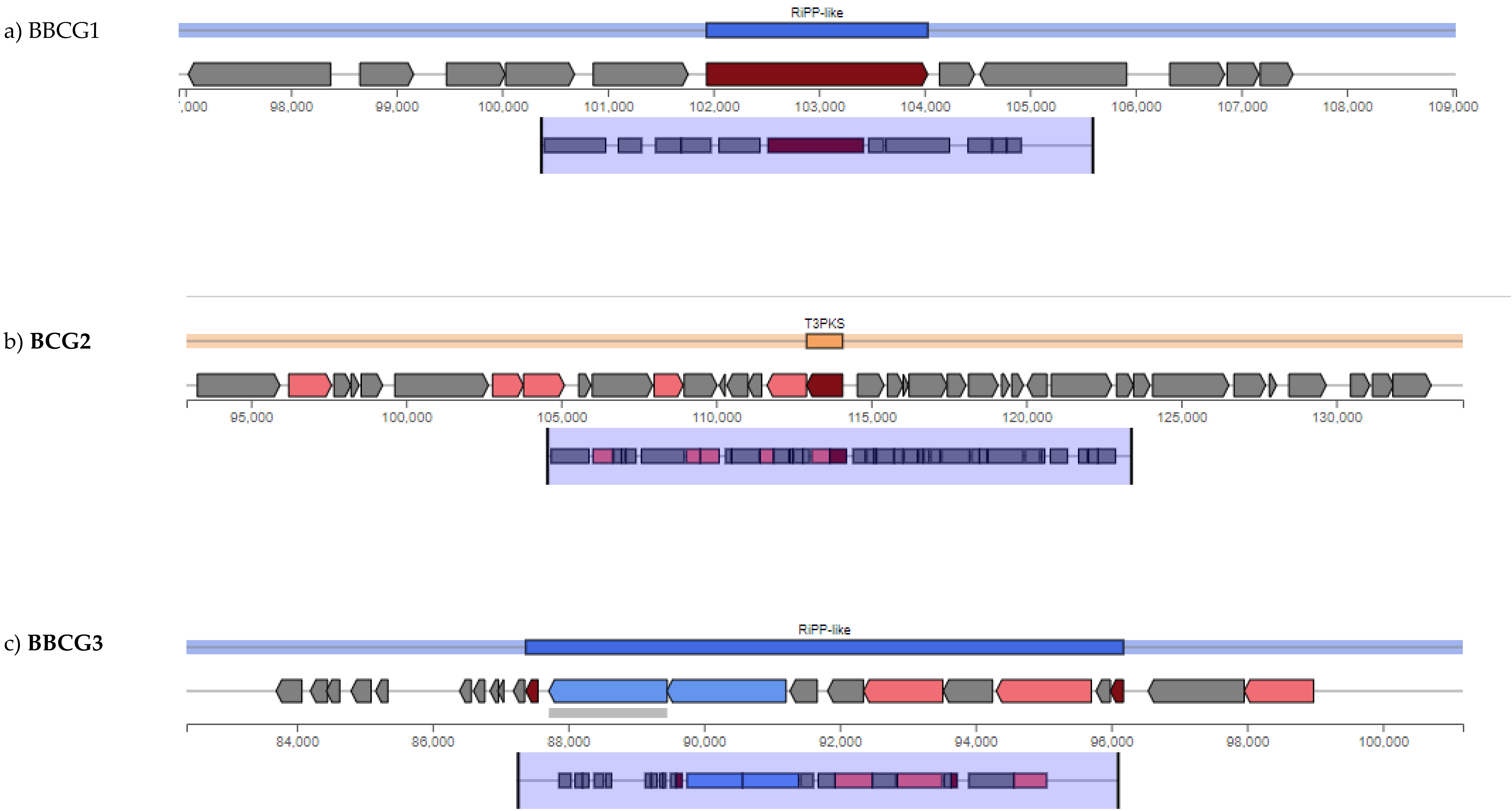

Figure S2: biosynthetic gene clusters (BGCs)identified in *S.salivarius* 24SMBc genome by antiSMASH. Core biosynthetic genes (brown); additional biosynthetic genes (salmon); transport- related genes (blue); other genes (grey)
