# Supplementary material for: Streptococcus salivarius 24SMBc Genome Analysis Reveals New Biosynthetic Gene Clusters Involved in Antimicrobial Effects on Streptococcus pneumoniae and Streptococcus pyogenes
Source: Microorganisms. 2022 Oct 16;10(10):2042. doi: 10.3390/microorganisms10102042 (PMC9610097; doi:10.3390/microorganisms10102042)
Supplement: Supplementary file 1 [file microorganisms-10-02042-s001.zip › Figure S3.pdf]

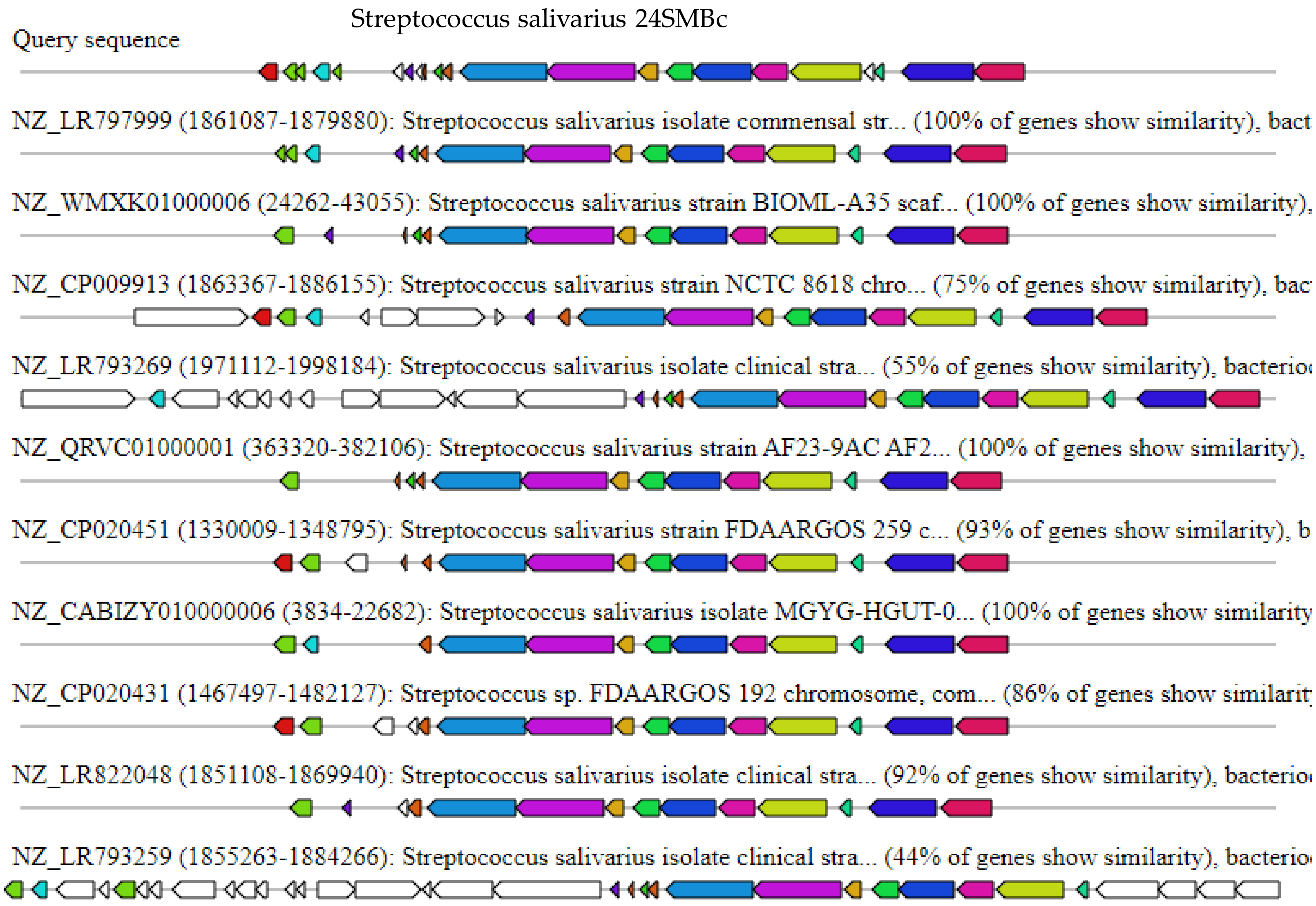

Figure S3: Cluster blast of BBCG3 by antiSMASH database. Cluster blast showed the top ten gene clusters that are most similar to BBCG3. Putative homologous genes were with the same color.
