# Supplementary material for: Streptococcus salivarius 24SMBc Genome Analysis Reveals New Biosynthetic Gene Clusters Involved in Antimicrobial Effects on Streptococcus pneumoniae and Streptococcus pyogenes
Source: Microorganisms. 2022 Oct 16;10(10):2042. doi: 10.3390/microorganisms10102042 (PMC9610097; doi:10.3390/microorganisms10102042)
Supplement: Supplementary file 1 [file microorganisms-10-02042-s001.zip › table S1.pdf]

	Assay condition	CFU/ml Pnc/Pyo (viable count)	% adherence (viable count)
Pnc alone (10^8 CFU/ml)	*S. pneumoniae BT*	1,30E+05	100
	S. pneumoniae C2	4,27E+05	100
	S. pneumoniae A3	4,77E+04	100
	S. pneumoniae M4	1,14E+05	100
Pnc (10^8) + *Sal* (10^9 CFU/ml)	*S. pneumoniae BT*	1,33E+04	9.88
	S. pneumoniae C2	1,37E+05	32.16
	S. pneumoniae A3	1,92E+04	40.31
	S. pneumoniae M4	4,00E+04	27.80
Pyo alone (10^8 CFU/ml)	*S. pyogenes 35370*	1,20E+06	100
	S. pyogenes 2812A	4,68E+06	100
	S. pyogenes 5005	4,83E+06	100
Pyo (10^8) + *Sal* (10^9 CFU/ml)	*S. pyogenes 35370*	1,12E+06	93.99
	S. pyogenes 2812A	4,30E+06	91.81
	S. pyogenes 5005	2,05E+06	42.41

Table S1. *S. pneumoniae* and *S. pyogenes* adherence to *HEp-2* cells determined by viable count. * Pnc = *S. pneumoniae*; Sal = *S. salivarius* 24SMBc and Pyo= *S. pyogenes*.
